# Aging attenuates the ovarian circadian rhythm

**DOI:** 10.1007/s10815-020-01943-y

**Published:** 2020-09-14

**Authors:** Ziru Jiang, Kexin Zou, Xia Liu, Hangchao Gu, Yicong Meng, Jing Lin, Weihui Shi, Chuanjin Yu, Li Jin, Li Wang, Xinmei Liu, Jianzhong Sheng, Hefeng Huang, Guolian Ding

**Affiliations:** 1 The International Peace Maternity and Child Health Hospital, School of Medicine, Shanghai Jiao Tong University, 910 Hengshan Road, Shanghai, 200030 China; 2 Shanghai Key Laboratory of Embryo Original Diseases, Shanghai, China; 3 Department of Pathology and Pathophysiology, School of Medicine, Zhejiang University, Hangzhou, China

**Keywords:** Circadian rhythm, Ovary, Age, Fertility

## Abstract

**Objective:**

To study the effect of aging on ovarian circadian rhythm.

**Design:**

Human and animal study.

**Setting:**

University hospital and research laboratory.

**Patients/animals:**

Human granulosa cells were obtained by follicular aspiration from women undergoing in vitro fertilization (IVF), and ovarian and liver tissues were obtained from female C57BL/6 mice.

**Intervention(s):**

None.

**Main outcome measure(s):**

Expression of circadian genes in young and older human granulosa cells and circadian rhythm in ovaries and livers of young and older mice.

**Result(s):**

All examined circadian clock genes in human granulosa cells showed a downward trend in expression with aging, and their mRNA expression levels were negatively correlated with age (*P* < 0.05). Older patients (≥ 40 years of age) had significantly reduced serum anti-Müllerian hormone (AMH) levels. Except for Rev-erbα, all other examined circadian clock genes were positively correlated with the level of AMH (*P* < 0.05). The circadian rhythm in the ovaries of older mice (8 months) was changed significantly relative to that in ovaries of young mice (12 weeks), although the circadian rhythm in the livers of older mice was basically consistent with that of young mice.

**Conclusion(s):**

Lower ovarian reserve in older women is partially due to ovarian circadian dysrhythmia as a result of aging.

**Electronic supplementary material:**

The online version of this article (10.1007/s10815-020-01943-y) contains supplementary material, which is available to authorized users.

## Introduction

The circadian clock is also known as the physiological clock. In mammals, the circadian clock system is composed of coordinated and synchronized cell and tissue clocks, including two main parts: the central clock located in the suprachiasmatic nucleus (SCN) of the basal hypothalamus and the peripheral clock in the various tissues of the body. Therefore, the internal circadian clock organization is broadly defined as a coordinated and synchronized central and peripheral clock rhythm [1]. Disruption of the circadian clock affects many physiological functions in the body and can result in, for example, sleep disorders, endocrine disorders, and decreased immune function and may even lead to certain diseases [2].

Circadian rhythms are produced at the molecular level by rhythmic transcription of clock genes. Most differentiated mammalian cells have a self-regulated transcription and translation feedback loop for clock gene transcription factors [3]. The transcription enhancers Bmal1 and Clock are the core of the time oscillator, and Clock shows histone acetyltransferase activity on its target promoters [4]. The Bmal1:Clock complex binds to the E-box sequences in the target promoters of periodic genes (Per) and cryptochrome genes (Cry) and drives their transcription. Concurrently, post-translational modifications of Per and Cry proteins inhibit the activity of Bmal1:Clock enhancer complexes and thus inhibit their own transcription. An intrinsic delay in the nuclear transfer of inhibitory complexes that results from similar phosphorylation and protein degradation pathways is the basis for the nearly 24 h of oscillator time. In addition to the above circuits, there is a secondary circuit of linked transcriptional regulators, including the inhibitory inhibitor receptor (Rev-erbα) and the enhancer retinoic acid-like orphan receptor α (ROR α), both of which maintain functional stability by regulating the expression of Bmal1 [5].

With the discovery of the ovarian circadian clock [6], the reproductive system circadian clock has become a new hotspot for research. The ovarian circadian clock is a peripheral circadian clock that is regulated by neuroendocrine signals from the central circadian clock, the SCN. The ovarian clock plays an important role in the physiological process of the normal reproductive system, such as ovulation [7] and steroid release [8]. Either a Bmal1 knockout or a Per1/Per2 mutation leads to reproductive disability in mice [8–11]. Coordination and synchronization of various circadian clocks on the hypothalamus-pituitary-ovary axis promote reproduction. Genetic polymorphism of the circadian clock leads to out-of-sync circadian clocks on the hypothalamic-pituitary-gonadal (HPG) axis, which may be one of the causes of some complex reproductive system diseases. Shift work, jet lag syndrome, sleep deprivation, and circadian clock gene knockout models that lead to changes in biological rhythms, in addition to dyssynchrony of circadian clocks on the hypothalamus-pituitary-ovary axis, can seriously affect reproductive function, including changes in hormone secretion patterns, decreased pregnancy rates, increased miscarriage rates, and increased risk of breast cancer [12].

It is generally believed that there is an interaction between the circadian clock and the aging process. In addition to a large body of heterogeneous and anecdotal literature, neuronal activity rhythms have been found to exhibit age-dependent degeneration in the SCN [13]. This decline in the central clock, which is associated with age, disrupts the ability of mice to respond to external light signals and alters circadian behavior [14]. However, the effect of aging on the ovarian clock has hardly been explored.

As women get older, the quality and quantity of their oocytes decline, as does the pregnancy rate for older women [15, 16]. Even with assisted reproductive techniques, such as in vitro fertilization and embryo transfer (IVF-ET), the pregnancy outcome is still poor [15]. Our previous study also suggested that older women may have lower fertility and lower live birth rates [16]. Because a decline in the ovarian reserve is one of the main reasons for the decline in fertility among older women, especially women after the age of 40, we investigated the expression of circadian genes in human granulosa cells from young and older women, and, the expression of circadian genes in ovarian tissues from young and older female mice to explore the effect of aging on the ovarian clock.

## Material and methods

### Subjects

We included the women with normal menstrual cycle, male factor infertility, or tubal factor infertility without hydrosalpinx, while excluded the patients with ovulation disorder, endometriosis, chromosome abnormality, and other infertility causes associated with ovarian dysfunction. A total of 52 patients who underwent IVF in the International Peace Maternity and Child Health Hospital, Shanghai Jiao Tong University School of Medicine, between September 2018 and January 2019 were finally included in this study. The patients were divided into two groups: the young group (< 40 years old) consisted of 34 individuals (25 tubal factor infertility and 9 male factor infertility), and the old group (**≥** 40 years old) consisted of 18 individuals (9 tubal factor infertility and 9 male factor infertility). The cause of infertility in these patients was tubal factors or male factors, without other complications. This study has been approved by the review committee of our hospital. All patients gave informed consent. We were permitted to use the patients’ data in the electronic database for research purposes, with the promise that the identities of the patients would remain anonymous and their medical records would remain confidential.

### Isolation of ovarian granulosa cells

Ovarian luteinized granulosa cells were obtained from each patient by follicular aspiration during the same period of the day (between 8 am and 10 am). All granulosa cells were collected from mature follicles, with a diameter > 14 mm as defined by ultrasound. About 10 ml of unpurified follicular fluid from each patient were placed in a 15-ml disposable sterile tube and centrifuged for 10 min at 400×*g*. After removal of the supernatant, the layers containing the granulosa cells along with the red blood cell pellet were gently layered on 8.0 ml of Ficoll-Paque Plus (Amersham Biosciences, Piscataway, NJ) in a 15-ml disposable sterile tube and centrifuged at 600×*g* for 20 min. The middle cell layer was gently transferred to a new 15-ml disposable sterile tube, washed three times with 10-ml phosphate-buffered saline, and centrifuged at 200×*g* for 5 min after each wash. All the purified cells from each participant were stored in 1-ml TRIzol (Thermo Fisher Scientific) at − 80 °C for RNA extraction.

### Animals

Female C57BL/6 mice were bred in the animal facility at the Shanghai Model Organisms Center under a 12-h light/12-h dark cycle (Zeitgeber time (ZT)0: 7 am light on; ZT12: 7 pm light off) with food and water ad libitum. Animals were group-housed with five mice per cage and allowed to acclimatize to the housing facility for at least 1 week. The temperature and humidity were kept at 20 °C to 24 °C and 55 ± 10%, respectively. The young group consisted of 12-week-old mice, and the old group consisted of 8-month-old mice. The stage of the estrous cycle was determined by cellular profile analysis of vaginal smears. During proestrus, three to five mice per group were sacrificed every fourth hour (ZT 0, ZT 4, ZT 8, ZT 12, ZT 16, ZT 20) to collect liver and ovarian tissues. All animal protocols were approved by the Institutional Animal Care and Use Committee of Shanghai Model Organisms Center (permit no. 2018-0035).

### Vaginal cytology method

A vaginal swab was collected using a cotton-tipped swab (Puritan Medical Products Company, LLC, Guilford, ME) wetted with ambient-temperature physiological saline and inserted into the vagina of the restrained mouse. The swab was gently turned and rolled against the vaginal wall and then removed. Cells were transferred to a dry glass slide by rolling the swab across the slide. The slide was air dried and then stained with ~ 400 mL of stain (Accustain, Sigma-Aldrich) for 45 s. The slides were rinsed with water, overlaid with a coverslip, and viewed immediately at ×20 magnification under bright-field illumination. The stage of the estrous cycle was determined based on the presence or absence of leukocytes and cornified epithelial and nucleated epithelial cells [17]. Results from vaginal smears of young and old mice are shown in Supplementary Figure 2.

### Real-time quantitative polymerase chain reaction

RNA was extracted from isolated human granulosa cells or mouse tissue using TRIzol (Takara) according to the manufacturer’s protocols. The purity and concentration of the RNA were determined from OD 260/280 readings by using a spectrophotometer (NanoDrop). For RT-PCR, 1 μg of total RNA was treated with DNase I to remove genomic DNA. Then first-strand cDNA was generated using 1 μg of denatured total RNA at 37 °C for 15 min, 84 °C for 5 s, and 4 °C for 5 min using a PrimeScript RT reagent kit with gDNA Eraser (Takara). qPCR was carried out in an ABI-Prism 7500 Sequence Detection System. According to the protocol, 1 μl cDNA was then used for PCR amplification of human and mouse circadian clock genes by denaturation at 95 °C for 5 s and annealing-extension at 60 °C for 34 s for 40 cycles using SYBR premix Ex Taq (Takara) and specific primers. The total reaction system of qPCR was 10 μl and the final concentration of primers was 1 μM. The 2^−ΔΔCT^ method was used to analyze the fold changes in expression of target genes relative to the internal control gene. Glyceraldehyde-3-phosphate dehydrogenase (GAPDH) served as an internal control. The specificity of qPCR products was confirmed by single peak melting curves in Supplementary Figure 3. No amplification of fragments occurred in negative control samples prepared without reverse transcriptase, or negative control samples prepared without template. For qPCR assay, three technical replicates were applied for each sample. The primer sequences are shown in Supplementary Table 1.

### Statistics

Data are presented as the mean ± SEM. Statistical analysis was performed by unpaired two-tailed Student’s *t* tests, one-way analysis of variance with post hoc tests, or their equivalent nonparametric tests (SPSS, version 24; IBM). *P* < 0.05 was considered statistically significant.

## Results

### Demographic data and clinic characteristics of young and older women

We collected granulosa cells from 34 young women and 18 older women and analyzed their clinical characteristics (Table 1). The body mass index (BMI) between the two groups did not differ statistically. Compared with young women, older patients had higher serum follicle stimulating hormone (FSH) levels. There were no differences in serum levels of luteinizing hormone (LH), prolactin (PRL), testosterone (T), progesterone (P), and estradiol (E2) between the two groups, even though the older group had a tendency toward a higher level of estrogen (163.97 ± 70.84 pmol/L and 204.94 ± 110.39 pmol/L, respectively). We also found that the older patients had significantly reduced serum AMH levels as compared with the young group (1.12 ± 0.67 ng/ml and 5.62 ± 3.77 ng/ml, respectively; *P* < 0.001).

**Table 1:** Demographic data and clinical characteristics of young and older women

Variable	Group		*P* value
	Age < 40 years old	Age ≥ 40 years old	
Cycles, *n*	34	18	
Age, years	28.65 ± 0.48	42.00 ± 0.35	< 0.001
BMI	21.79 ± 0.62	22.42 ± 0.76	0.551
FSH, IU/L	7.67 ± 0.25	9.99 ± 0.94	0.037
LH, IU/L	4.80 ± 0.43	3.95 ± 0.26	0.111
LH/FSH	0.63 ± 0.05	0.46 ± 0.05	0.062
PRL, μg/L	24.52 ± 6.62	11.91 ± 1.02	0.247
E_2_, pmol/L	163.97 ± 11.96	204.94 ± 25.24	0.178
T, nmol/L	2.62 ± 1.10	0.91 ± 0.10	0.334
P, nmol/L	1.42 ± 0.12	2.33 ± 0.20	0.287
AMH, ng/mL	5.62 ± 0.54	1.12 ± 0.14	< 0.001

Values are presented as the *n*, mean ± SEM, or ratio. *BMI*, body mass index; *FSH*, follicle stimulating hormone; *E*_*2*_, estradiol; *LH*, luteinizing hormone; *PRL*, prolactin; *T*, testosterone; *P*, progesterone; *AMH*, anti-Müllerian hormone

### Expression of circadian clock genes in granulosa cells from young and older women

**Fig. 1:**
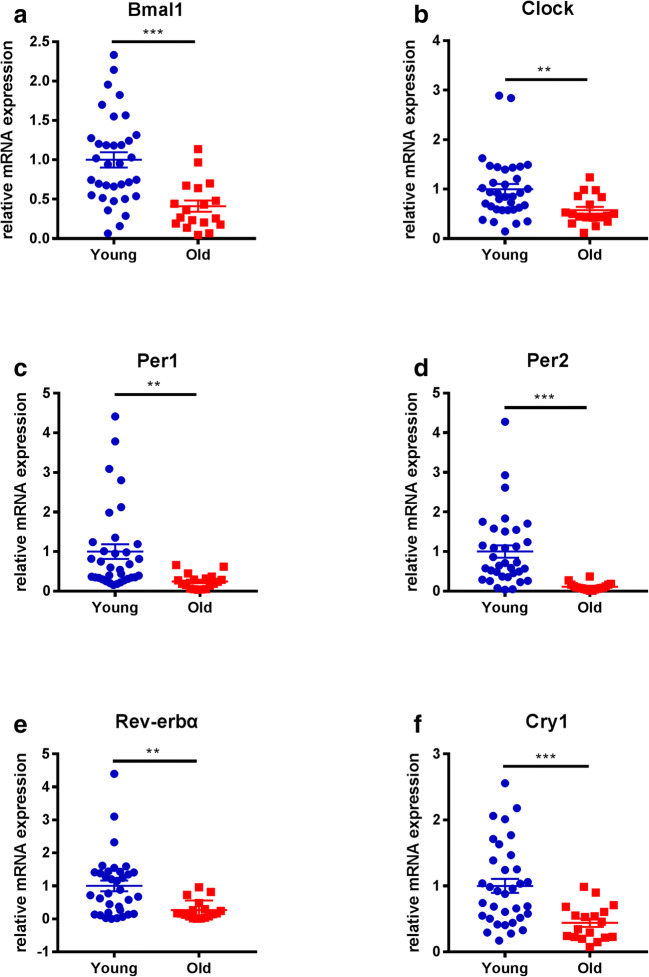
Relative mRNA expression of circadian clock genes in granulosa cells from young and older women. Young group, < 40 years old (*n* = 34); older group, ≥ 40 years old (*n* = 18). All data were expressed as the mean ± SEM. **P < 0.01; ***P < 0.001

**Fig. 2:**
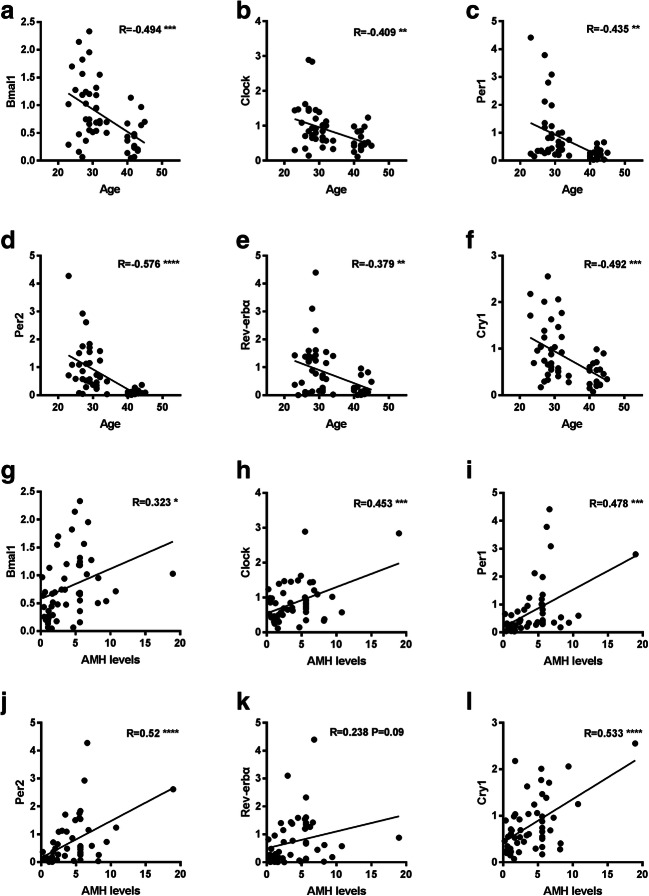
Correlation between the expression of circadian clock genes in granulosa cells and the age or AMH level of the individual. **a–f** Correlation between the expression of circadian clock genes in granulosa cells and age (*n* = 52). **g–l** Correlation between the expression of circadian clock genes in granulosa cells and AMH (*n* = 52). **P < 0.01; ***P < 0.001; ****P < 0.0001

All examined circadian clock genes tended to decrease in expression with aging, and these differences were all statistically significant (Fig. 1). We also examined a marker of differentiation (LHCGR) and another internal reference (β-actin) in luteinized granulosa cells of young and older women. There was no significant difference in the expression of LHCGR or β-actin between the two groups (Supplementary Figure 1). The correlation between the expression of circadian clock genes and age or AMH level was analyzed. The mRNA expression levels of the circadian genes Bmal1, Per1, Per2, Rev-erbα, Clock, and Cry1 were all negatively correlated with age (*P* < 0.05) (Fig. 2a–f). Except for Rev-erbα, all other circadian clock genes were positively correlated with the level of AMH (*P* < 0.05) (Fig. 2g–l). We also compared the correlation of the genes to FSH and estradiol but finding no obvious correlation (data not shown).

### Rhythmic expression of circadian clock genes in ovaries of young and old female mice

**Fig. 3:**
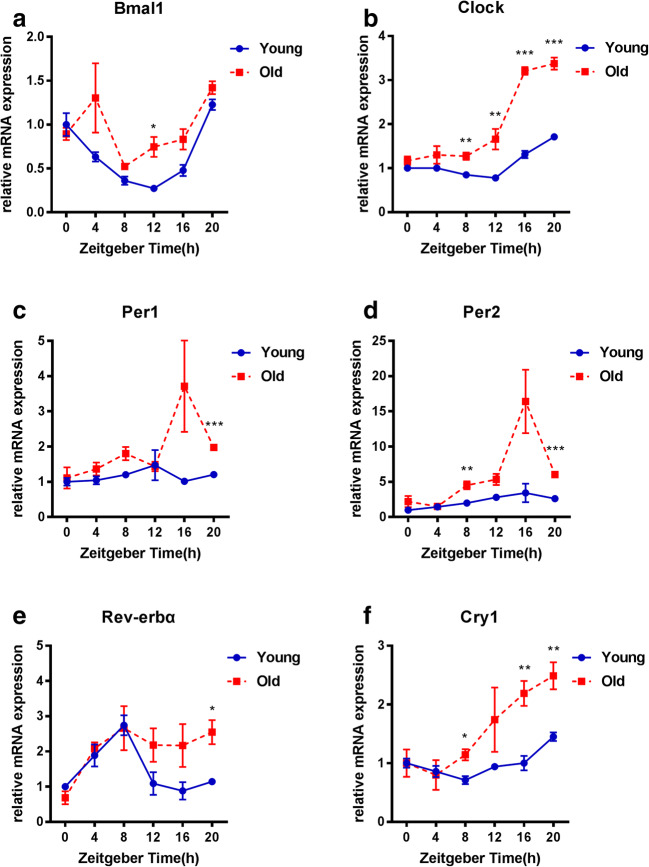
Rhythmic expression of circadian clock genes in ovaries of young and older female mice. Young group, 12-week-old mice (*n* = 3–7 mice per time point); old group, 8-month-old mice (*n* = 3–7 mice per time point). All data are expressed as the mean ± SEM. *P < 0.05; **P < 0.01; ***P < 0.001

In the female mouse, the estrous cycle is divided into four stages (proestrus, estrus, metestrus, and diestrus) and repeats every 4 to 5 days unless interrupted by pregnancy, pseudopregnancy, or anestrus. Vaginal smears showed that there were four stages in both young (12-week-old) and old (8-month-old) female mice (Supplementary Figure 2). The expression pattern of circadian clock genes in the ovaries of young mice showed obvious diurnal variation. As shown in Fig. 3, in the young mice, the expression levels of Bmal1, Clock, and Cry1 increased at night (ZT12-20) and decreased during the day (ZT0-12), whereas the trends for Per1, Per2, and Rev-erbα were opposite. Compared with the young mice, the older mice showed a significant change in the rhythm of circadian clock gene expression in their ovaries. First, gene expression levels changed exponentially at specific time points. Second, the timing of the peak or valley of the expression of individual genes shifted in the older mice relative to that in the young mice.

For comparison, we detected the rhythmic expression of circadian clock genes in the livers of these young and older mice. We found that the circadian rhythm of the circadian clock genes in the livers of older mice was consistent with that of young mice (Supplementary Figure 4).

## Discussion

Ovarian age is an independent variable that strongly affects IVF outcome. Low functional ovarian reserve can develop physiologically with advancing reproductive age. Women generally reach peak reproductive potential well before the age of 30 and then experience a slow decline in reproductive potential until the age of 40, at which point the decline becomes more precipitous [18]. Although IVF-ET is the most effective treatment for women who are > 40 years old [15], the success rate for older women is still very low [16]. It is commonly known that fertility decreases with advancing age. Consistently, it is estimated that 13 years before menopause, even in the presence of a regular menstrual cycle, the ovaries begin to signal an accelerated decline in fertility [19]. In assisted reproductive techniques, women who achieve a live birth are significantly younger and produce more oocytes and transferable embryos than those who do not [20]. In our previous study, we found that, compared with young patients, older patients showed significantly decreased numbers of retrievable oocytes and available embryos and had a lower clinical pregnancy rate after assistant reproductive technology. In addition, we compared the final outcomes between young and old pregnant women and found that older women have significantly increased miscarriage rates and decreased live birth rates [16].

The process of aging and circadian rhythms are intimately intertwined, and changes in central rhythmic behavior are associated with age-dependent decline [21]. However, how aging influences circadian rhythms and how the clock contributes to the aging process are unresolved questions. The rhythm of neuronal activity shows age-related degeneration in the SCN, where the central circadian pacemaker is located [13]. Because of this decline in the central clock that is associated with aging, the physiological ability of mice to adapt to an external light signal is disrupted, which changes their rhythmic behavior [14, 22]. In addition, a genetic mouse model has shown that deficiency in the core circadian transcription factor aryl hydrocarbon receptor nuclear-translocator-like (Arntl or Bmal1) results in a reduced lifespan and a premature aging phenotype in mice [23].

Normal reproductive function requires an ovarian circadian clock. The reproductive system of female mammals shows a precise rhythm. In mammals, circulating gonadotropin LH and FSH levels oscillate with a diurnal rhythm marked by significant afternoon “surges” on the day of ovulation [24]. Therefore, the ovarian circadian clock may dominate the rhythmic expression of key players in the ovulatory response pathway. Rhythmic expression of clock genes has been reported in intact ovaries and isolated granulosa cells from both rats and mice [6, 11, 25]. It is possible that the ovarian clock modulates the timing of ovulation by regulating the expression of gonadotropin receptors and/or regulates the timing and amplitude of steroid and peptide hormone secretion. It has been suggested that the timing of ovulation may depend on rhythmic sensitivity of the ovary to gonadotropins [7]. Mice with a conditional knockout of the Bmal1 locus in steroidogenic cells (steroidogenic factor 1 [SF1]-driven Cre in Bmal1^flox/flox^ mice) showed severe deficits in implantation success and compromised progesterone secretion [26]. Normal implantation was rescued when wild-type ovaries were transplanted into SF1-Cre;Bmal1^−/−^ mice, and implantation rates were reduced when, conversely, knockout ovaries were transplanted into wild-type hosts. These data strongly suggest that the ovarian clock plays a role in fertility, linked in large part to the control of ovarian steroid hormone synthesis.

Women over the age of 40 years may not fall into the category of “old age,” but they have reached an age that is relevant for the female reproductive system and fertility. As a result, the circadian clock of women after the age of 40 may have different effects on different organs. Clock genes such as Bmal1, Per1, Per2, Rev-erbα, Clock, and Cry1 are expressed in human granulosa cells. At present, however, few studies have focused on the effect of aging on the circadian clock of human ovaries. Only one previous study has shown a decrease in clock gene expression in granulosa cells from older women, but the sample size was small (only five young women and five older women) and there was no strong evidence [27]. Hence, we have tried to study whether ovarian aging is related to the decreased expression of clock genes. Our results showed that the expression of clock genes was significantly decreased in the ovaries of elderly women relative to young women. There was no difference between the two groups in the expression of the differentiation marker LHCGR and the expression of the internal reference β-actin, suggesting that the decreased expression of clock genes was indeed related to age but was not related to a reduction in the extent of granulosa cell differentiation. In conclusion, ovarian clock decline may contribute to lower ovarian reserve in older women, and hormone levels may be one of the links between the decline of the ovarian clock and lower ovarian reserve.

Our findings from human granulosa cells suggested a difference in the expression of circadian clock genes at certain time points, but the effect of aging on the rhythm of the ovarian clock could not be verified in human samples, so we carried out mouse experiments. The expression of core clock genes and clock-controlled genes (CCGs) as well as the expression of core clock proteins in the liver is virtually unaltered when young and old mice that have been fed a normal diet are compared [28]. Similarly, we analyzed liver mRNAs and found that the rhythm of expression of circadian clock genes in the livers of 8-month-old mice was basically the same as that of 12-week-old mice. However, the results of the ovarian experiment were different. Compared with young mice, the relative mRNA gene expression in the old mice appears higher for most time points. Because of the change of gene expression in multiple time points, the overall rhythm changes. This dynamic response in the circadian clock genes found in the old mice indicated the sensitivity of the ovarian biological clock to aging. Besides the relative mRNA gene expression at each time point, we focused far more on the rhythm of the expression of the circadian clock gene. As expected, the circadian rhythm of the old mice was significantly different from that of the young mice. These results suggested the circadian clockwork in ovarian cells may be altered with aging, and, expression of clock genes at each time point may be related to the switch-on and switch-off of the circadian oscillation. Changes in gene expression at some time points resulted in the delay or advance of peak and trough of circadian rhythm. Thus aging is closely related to the ovarian circadian clock thus may affect ovarian function and lead to a decrease in ovarian reserve. Further study is needed to explore the specific molecular mechanism.

## Conclusion

Low functional ovarian reserve can develop physiologically with advancing reproductive age, thus may result in poor fertility outcomes even with assisted reproductive techniques. In our study, we found that lower ovarian reserve in older women may be partly due to ovarian circadian dysrhythmia as a result of aging. More studies will be needed to explain mechanistically how aging affects the circadian clock of the ovary.

## Electronic supplementary material

ESM 1 *ACTB* and *LHCGR* expression in granulosa cells from young and older women. Young group, <40 years old (*n* = 34); old group, **≥**40 years old (*n* = 18). All data are expressed as the mean ± SEM (PNG 208 kb)

High Resolution image (TIF 592 kb)

ESM 2 Vaginal smear of young and old female mice. Figure 2-A and Figure 2-B represent estrous cycles for young and old mice, respectively. The estrous cycle consists of four stages: proestrus, estrus, metestrus, and diestrus. Scale bar= 100 μm. (PNG 4024 kb)

High Resolution image (TIF 132674 kb)

ESM 3 Melt curve plot of qPCR. Amplification specificities of candidate reference gene primers in qPCR. (A–C) Melt curve analysis of circadian clock genes, LHCGR and GAPDH in Human granulosa cells. (D–G) Melt curve analysis of circadian clock genes and GAPDH in mouse liver and ovarian tissues. (PNG 2006 kb)

High Resolution image (TIF 2700 kb)

ESM 4 Rhythmic expression of circadian clock genes in livers from young and old female mice. Young group, 12-week-old mice (n = 3-7 mice per time point); old group, 8-month-old mice (n = 3-7 mice per time point). All data are expressed as the mean ± SEM.*P < 0.05; **P < 0.01. (PNG 355 kb)

High Resolution image (TIF 752 kb)

ESM 5 Primers for real-time qPCR (DOCX 16 kb)
