# Supplementary figures and images for: Aging attenuates the ovarian circadian rhythm
Source: J Assist Reprod Genet. 2020 Sep 14;38(1):33–40. doi: 10.1007/s10815-020-01943-y (PMC7822988; doi:10.1007/s10815-020-01943-y)

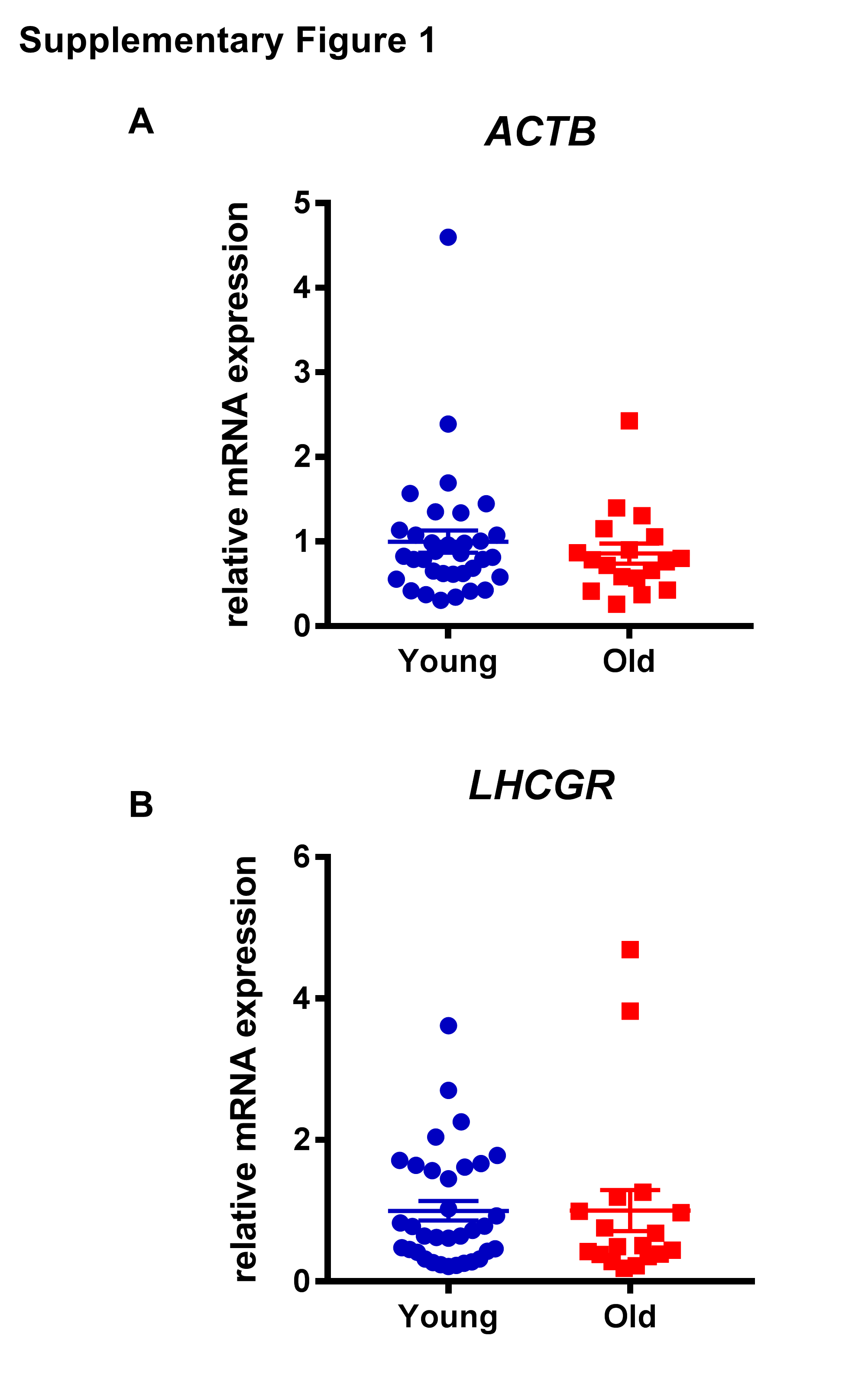

Supplement: Supplementary file 1 — ACTB and LHCGR expression in granulosa cells from young and older women. Young group, <40 years old (n = 34); old group, ≥40 years old (n = 18). All data are expressed as the mean ± SEM (PNG 208 kb) [file 10815_2020_1943_Fig4_ESM.png]

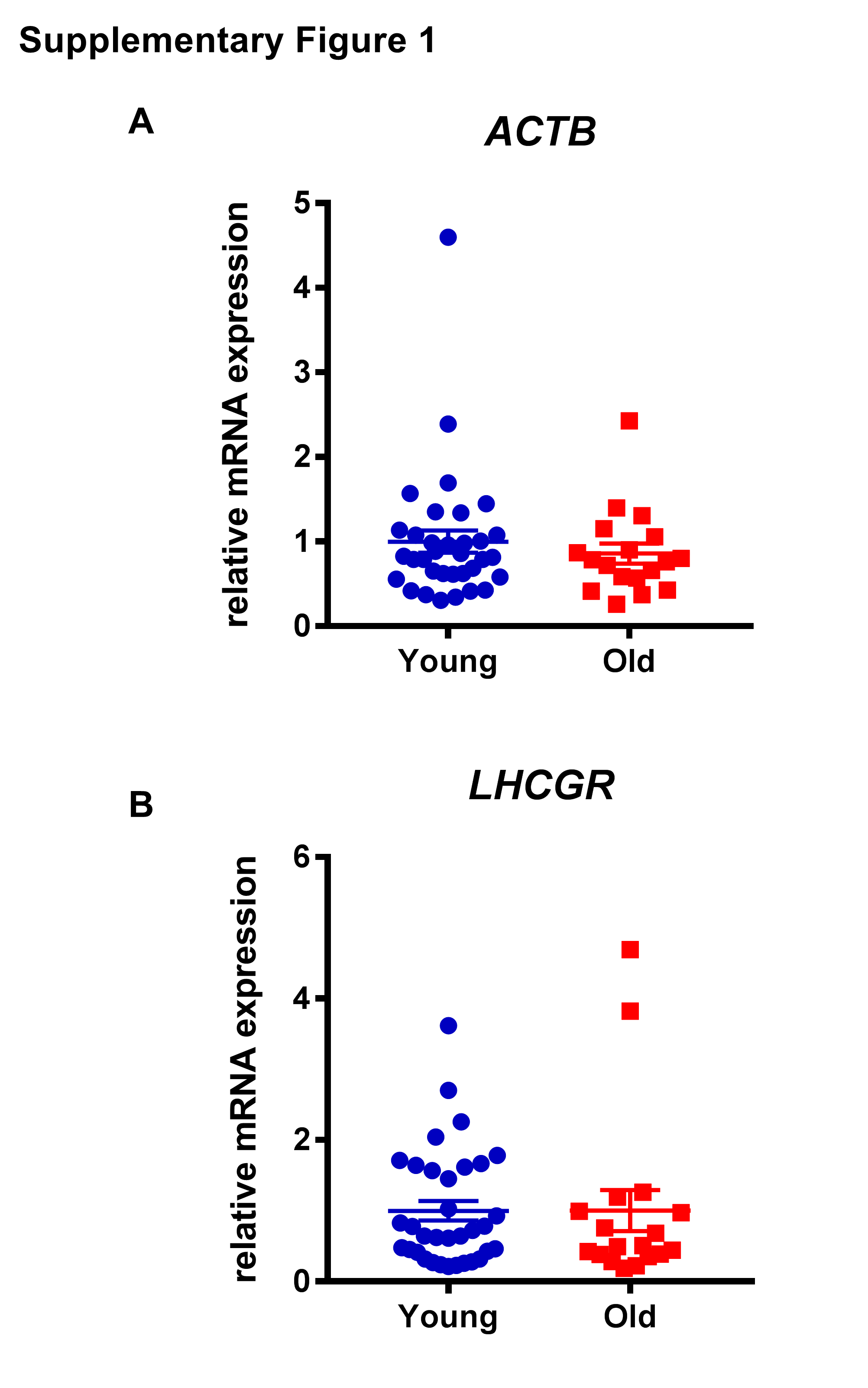

Supplement: Supplementary file 2 — High Resolution image (TIF 592 kb) [file 10815_2020_1943_MOESM1_ESM.tif]

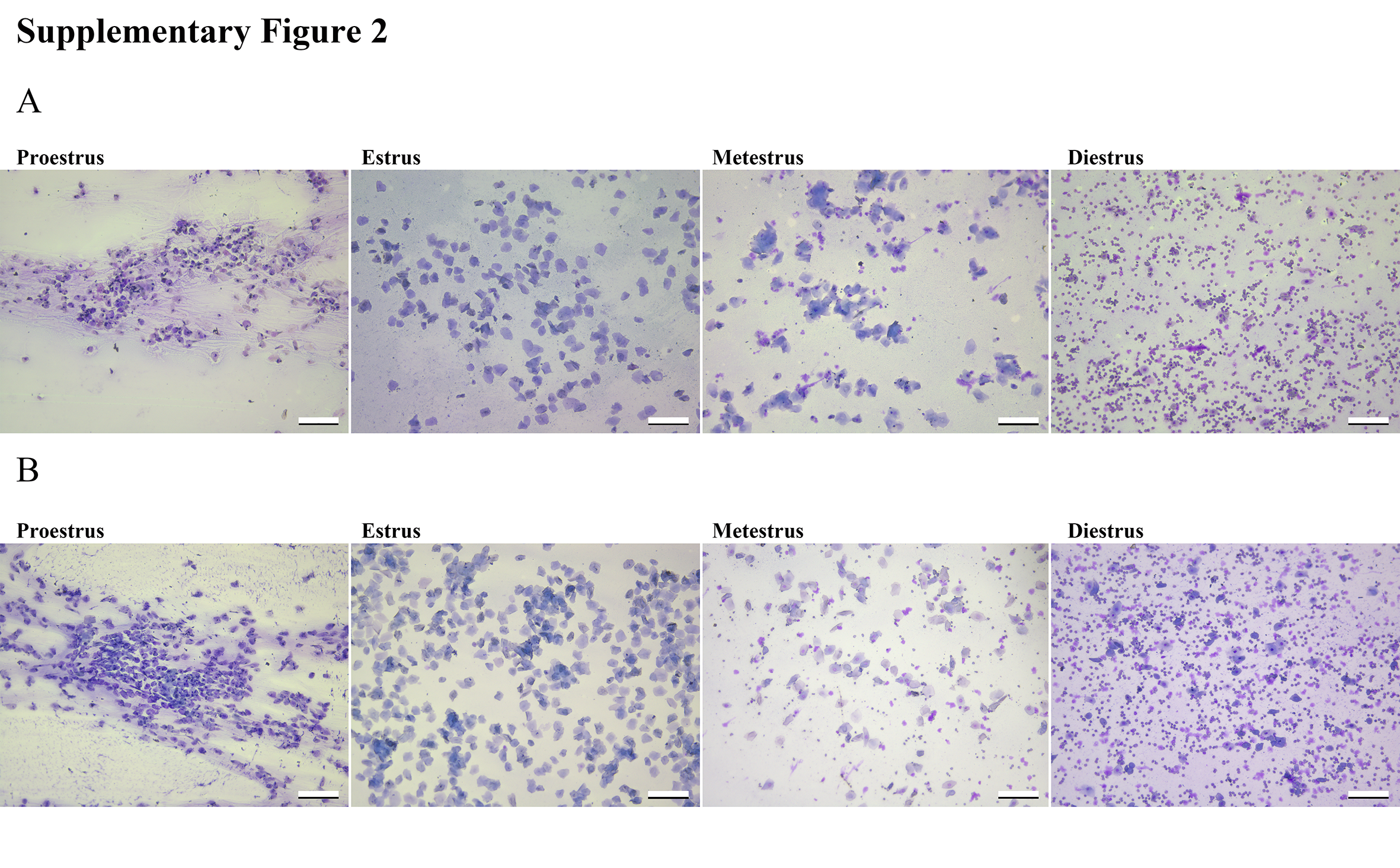

Supplement: Supplementary file 3 — Vaginal smear of young and old female mice. Figure 2-A and Figure 2-B represent estrous cycles for young and old mice, respectively. The estrous cycle consists of four stages: proestrus, estrus, metestrus, and diestrus. Scale bar= 100 μm. (PNG 4024 kb) [file 10815_2020_1943_Fig5_ESM.png]

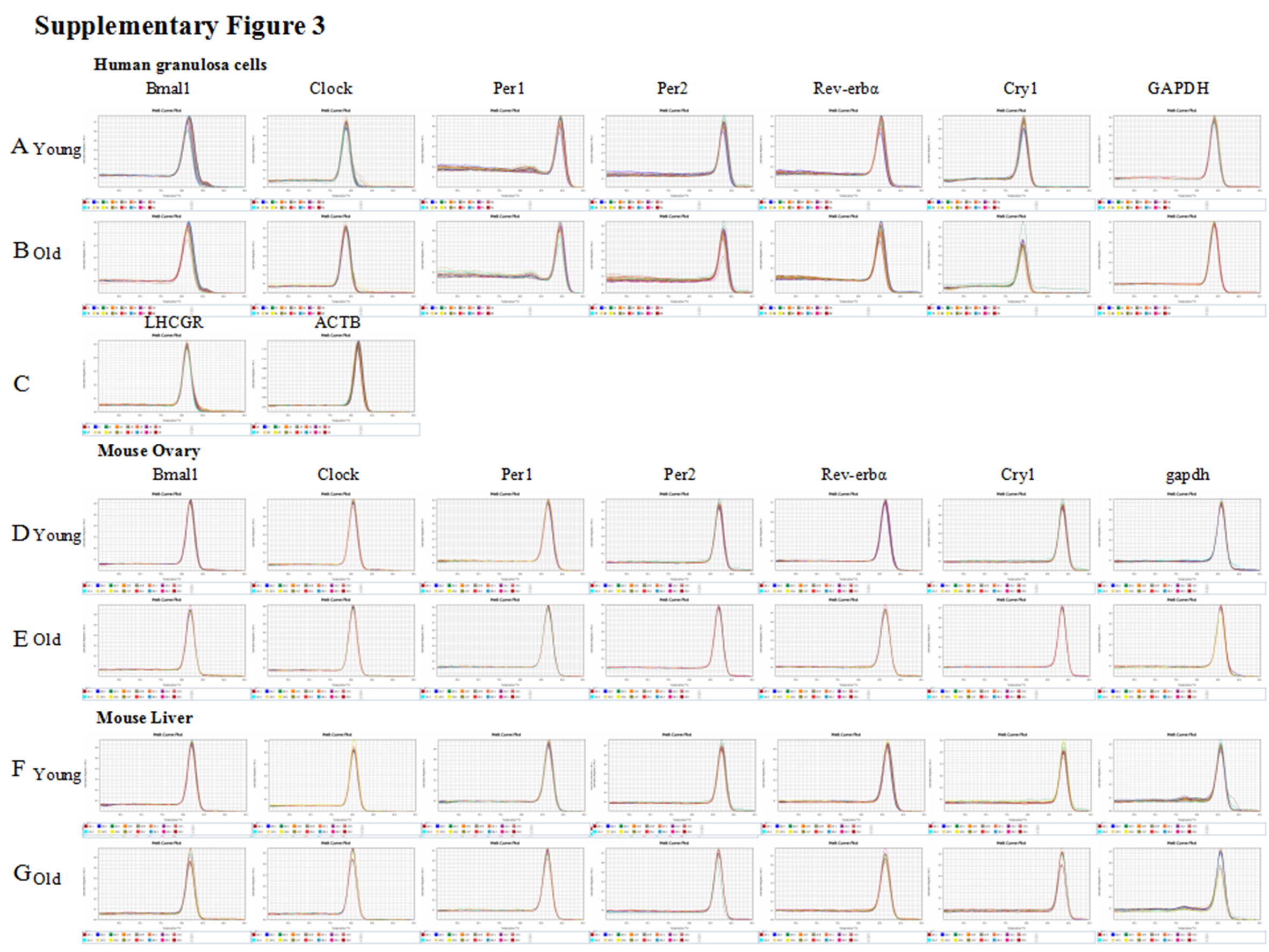

Supplement: Supplementary file 5 — Melt curve plot of qPCR. Amplification specificities of candidate reference gene primers in qPCR. (A–C) Melt curve analysis of circadian clock genes, LHCGR and GAPDH in Human granulosa cells. (D–G) Melt curve analysis of circadian clock genes and GAPDH in mouse liver and ovarian tissues. (PNG 2006 kb) [file 10815_2020_1943_Fig6_ESM.png]

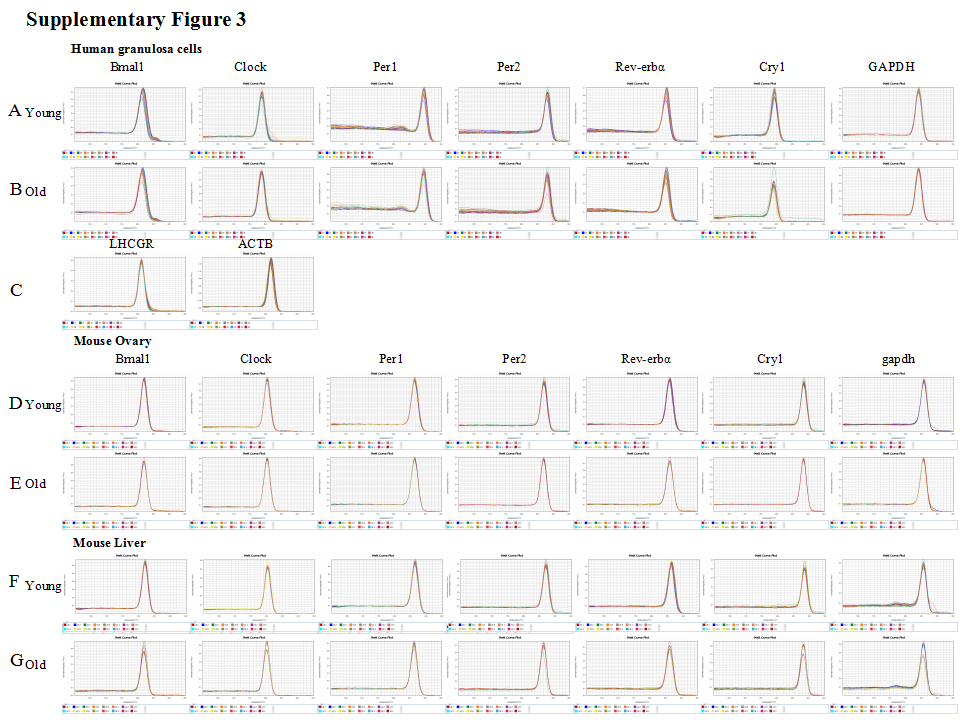

Supplement: Supplementary file 6 — High Resolution image (TIF 2700 kb) [file 10815_2020_1943_MOESM3_ESM.tif]

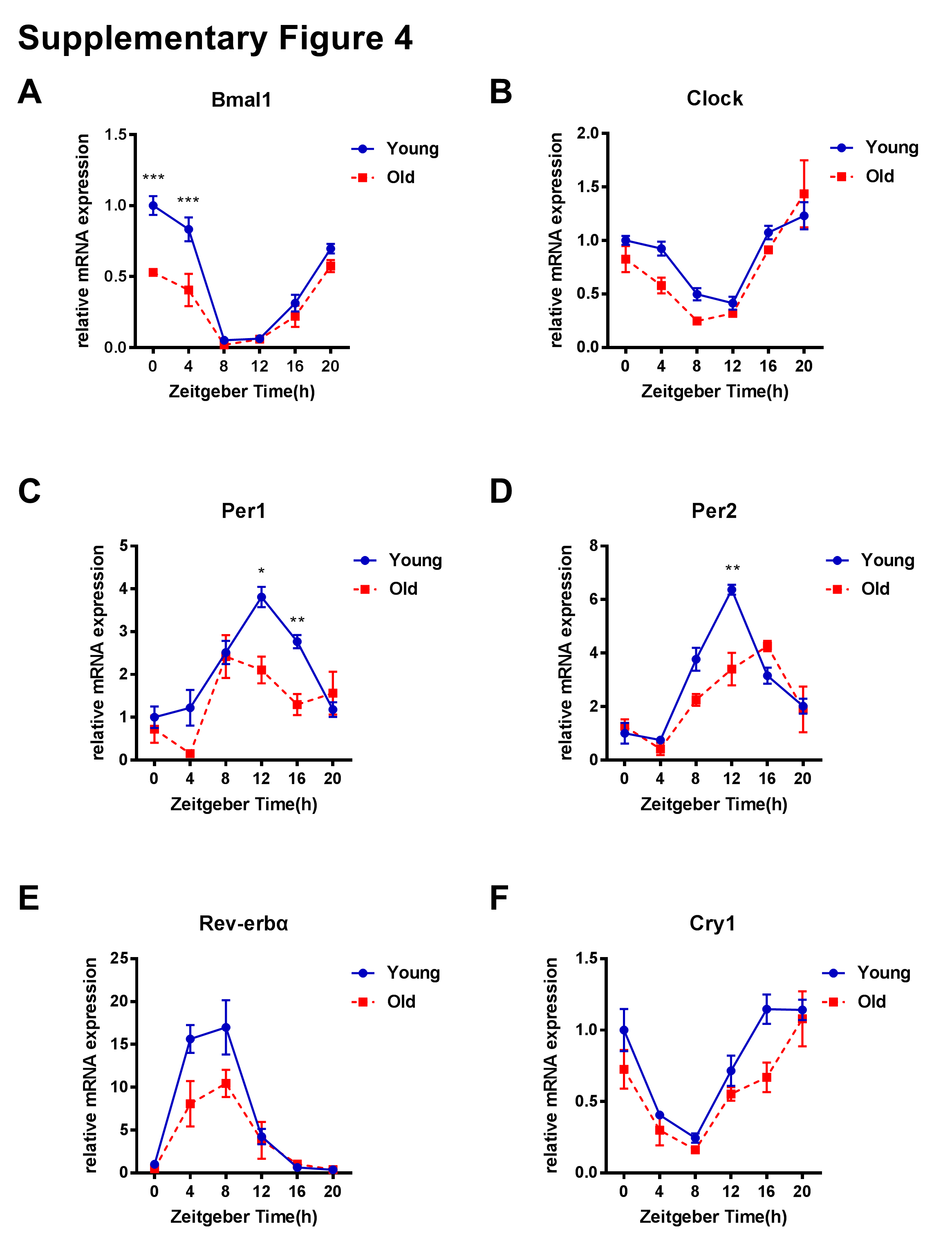

Supplement: Supplementary file 7 — Rhythmic expression of circadian clock genes in livers from young and old female mice. Young group, 12-week-old mice (n = 3-7 mice per time point); old group, 8-month-old mice (n = 3-7 mice per time point). All data are expressed as the mean ± SEM.*P < 0.05; **P < 0.01. (PNG 355 kb) [file 10815_2020_1943_Fig7_ESM.png]

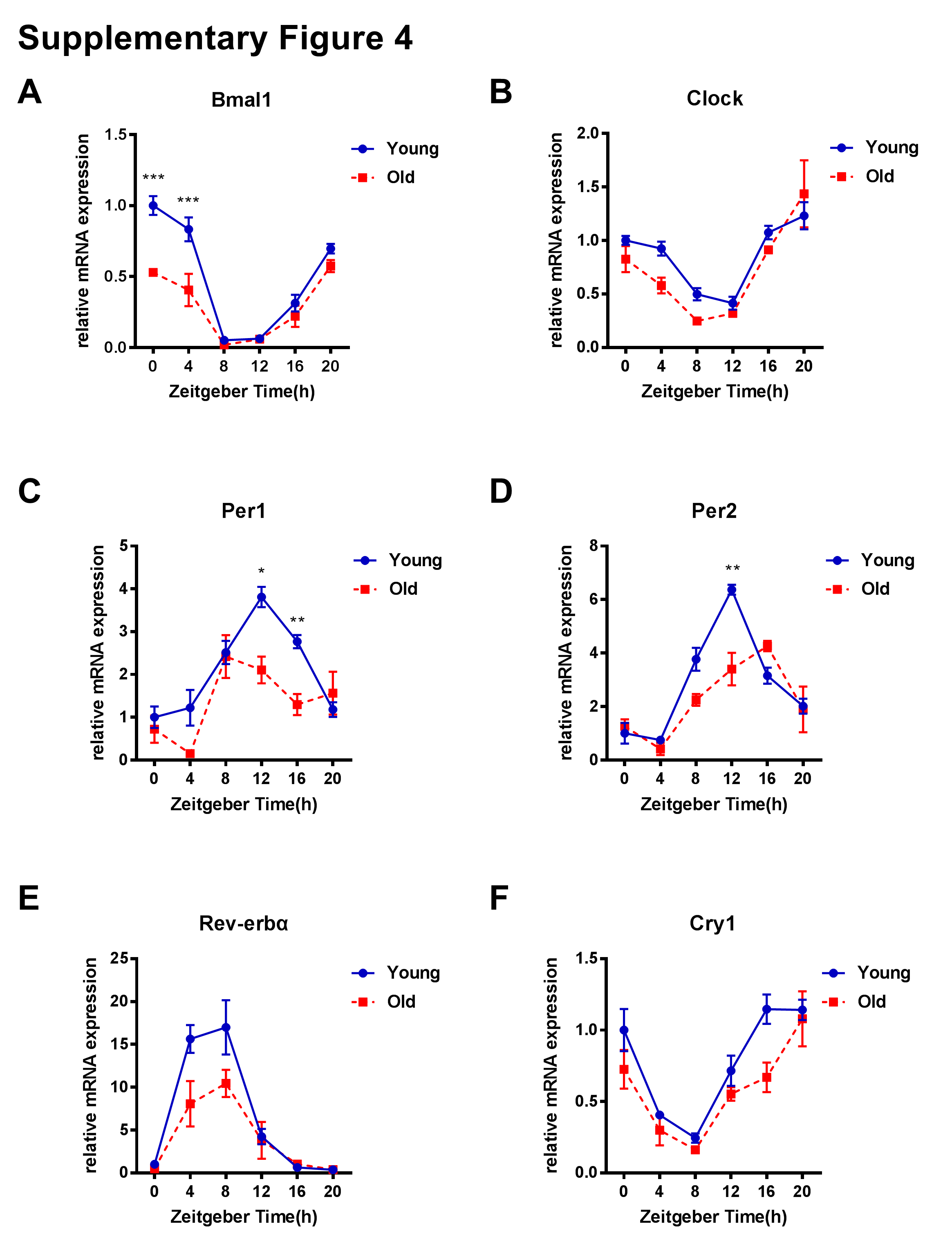

Supplement: Supplementary file 8 — High Resolution image (TIF 752 kb) [file 10815_2020_1943_MOESM4_ESM.tif]
