# Supplementary material for: Aging attenuates the ovarian circadian rhythm
Source: J Assist Reprod Genet. 2020 Sep 14;38(1):33–40. doi: 10.1007/s10815-020-01943-y (PMC7822988; doi:10.1007/s10815-020-01943-y)
Supplement: Supplementary file 9 — Primers for real-time qPCR (DOCX 16 kb) [file 10815_2020_1943_MOESM5_ESM.docx]

Supplementary Table1. Primers for real-time qPCR

| Species | Gene | Accession No. | Sequence 5'-3' | Amplicon (bp) |
| --- | --- | --- | --- | --- |
| Human | Clock | NM_001267843 | F: TGCGAGGAACAATAGACCCAA  R: ATGGCCTATGTGTGCGTTGTA | 138 |
| Human | Bmal1 | NM_001178 | F: CCGTGGACCAAGGAAGTAGA  R: CTGTGAGCTGTGGGAAGGTT | 97 |
| Human | Per1 | NM_002616 | F: GCAGGCTTCGTGGGCTTGACA  R: ATCGGCAGTGGTGTCGGCGA | 105 |
| Human | Per2 | NM_022817 | F: GACATGAGACCAACGAAAACTGC  R: AGGCTAAAGGTATCTGGACTCTG | 130 |
| Human | Cry1 | NM_004075 | F: TTGGAAAGGAACGAGACGCAG  R: CGGTTGTCCACCATTGAGTT | 125 |
| Human | Rev-erbα | NM_021724 | F: ACAGCTGACACCACCCAGATC  R: CATGGGCATAGGTGAAGATTTCT | 101 |
| Human | GAPDH | NM_002046 | F: ACCCACTCCTCCACCTTTGA  R: TCCACCACCCTGTTGCTGTA | 110 |
| Human | LHCGR | NM_000233 | F: GGTCTCACTCGACTATCACTTGC  R: CTCCGGGCTCAATGTATCTCA | 205 |
| Human | ACTB | NM_001101 | F: GCATCCCCCAAAGTTCACAA  R: AGGACTGGGCCATTCTCCTT | 153 |
| Mouse | Clock | [NM_007715](http://www.ncbi.nlm.nih.gov/entrez/query.fcgi?cmd=Search&db=Nucleotide&term=NM_007715) | F: ATGGTGTTTACCGTAAGCTGTAG  R: CTCGCGTTACCAGGAAGCAT | 197 |
| Mouse | Bmal1 | [NM_007489](http://www.ncbi.nlm.nih.gov/entrez/query.fcgi?cmd=Search&db=Nucleotide&term=NM_007489) | F: TGACCCTCATGGAAGGTTAGAA  R: GGACATTGCATTGCATGTTGG | 154 |
| Mouse | Per1 | [NM_001159367](http://www.ncbi.nlm.nih.gov/entrez/query.fcgi?cmd=Search&db=Nucleotide&term=NM_001159367) | F: CGGATTGTCTATATTTCGGAGCA  R: TGGGCAGTCGAGATGGTGTA | 142 |
| Mouse | Per2 | [NM_011066](http://www.ncbi.nlm.nih.gov/entrez/query.fcgi?cmd=Search&db=Nucleotide&term=NM_011066) | F: GAAAGCTGTCACCACCATAGAA  R: AACTCGCACTTCCTTTTCAGG | 186 |
| Mouse | Cry1 | [NM_007771](http://www.ncbi.nlm.nih.gov/entrez/query.fcgi?cmd=Search&db=Nucleotide&term=NM_007771) | F: CACTGGTTCCGAAAGGGACTC  R: CTGAAGCAAAAATCGCCACCT | 153 |
| Mouse | Rev-erbα | [NM_145434](http://www.ncbi.nlm.nih.gov/entrez/query.fcgi?cmd=Search&db=Nucleotide&term=NM_145434) | F: TACATTGGCTCTAGTGGCTCC  R: CAGTAGGTGATGGTGGGAAGTA | 127 |
| Mouse | Gapdh | NM_001289726 | F: GGAGAGTGTTTCCTCGTCCC  R: ATGAAGGGGTCGTTGATGGC | 136 |
